# Inosine monophosphate dehydrogenase type1 sustains tumor growth in hepatocellular carcinoma

**DOI:** 10.1002/jcla.24416

**Published:** 2022-04-11

**Authors:** Xiaoyuan Jia, Yao Liu, Yan Cheng, Yin Wang, Hui Kang, Zhongren Ma, Kan Chen

**Affiliations:** ^1^ 12646 College of Life Sciences and Medicine Zhejiang Sci‐Tech University Hangzhou China; ^2^ Department of Oncology Baoji Hi‐Tech Hospital Baoji China; ^3^ Gansu Tech Innovation Center of Animal Cell Biomedical Research Center Northwest Minzu University Lanzhou China; ^4^ Shaoxing Biomedical Research Institute Co. LTD Zhejiang Sci‐Tech University Shaoxing China

**Keywords:** animal model, hepatocellular carcinoma, inosine monophosphate dehydrogenase, transcriptomic profiling

## Abstract

**Background:**

Inosine monophosphate dehydrogenase (IMPDH) is the key enzyme in the biosynthesis of purine nucleotides. IMPDH1 and IMPDH2 are the two isoforms of IMPDH and they share 84% amino acid similarity and virtually indistinguishable catalytic activity. Although high expression of IMPDH2 has been reported in various cancers, the roles of IMPDH1 in hepatocellular carcinoma (HCC) are largely unknown.

**Methods:**

The expression and the clinical relevance of IMPDH1 in 154 HCC patients were detected by immunohistochemistry analysis. The stable IMPDH1 knockdown HuH7 cells were established by lentiviral RNAi approach. The single cell proliferation was detected by colony‐forming unit assay. The tumor initiation and growth ability were measured by using xenograft tumor model in immunodeficient mice. The effect of IMPDH1 on cellular signaling pathways was analyzed by genome‐wide transcriptomic profiling.

**Results:**

The expression of IMPDH1 is upregulated in tumor tissue compared with adjacent liver tissue, and higher expression of IMPDH1 is associated with better patient cumulative survival. In experimental models, loss of IMPDH1 in HCC cells inhibits the ability of single cell colony formation in vitro, and reduces the efficiency of tumor initiation and growth in immunodeficient mice. Consistently, loss of IMPDH1 results in distinct alterations of signaling pathways revealed by genome‐wide transcriptomic profiling.

**Conclusion:**

IMPDH1 sustains HCC growth and progression.

## Graphical abstract

We observed that the expression of IMPDH1 is upregulated in tumor tissue compared with adjacent liver tissue in 154 HCC patients, and higher expression of IMPDH1 is associated with better patient cumulative survival in our study. Loss of IMPDH1 in HCC cells inhibits the ability of single cell colony formation in vitro, and reduces the efficiency of tumor initiation and growth in immunodeficient mice. Transcriptomic analysis shows that loss of IMPDH1 results in distinct alterations of signaling pathways.

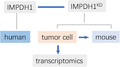

## 1 INTRODUCTION

Reprogramming of cellular metabolism is one of the key hallmarks of cancer,^1^ and this altered metabolism appears to be a direct consequence of oncogenic mutation.^2^ In addition, metabolic enzymes themselves are frequently mutated or amplified during tumorigenesis, which has been directly linked to disease progression.^3^, ^4^ Especially, the roles of hexokinase 2 (HK2), lactate dehydrogenase A (LDHA), and pyruvate dehydrogenase kinase 1 (PDK1) have been extensively studied in various cancers.^5^ However, for many enzymes of cellular metabolism, their roles in the carcinogenesis remain largely elusive.

The liver plays prominent roles in metabolism, and hepatocytes constitute biochemically the most active cells of the body. Thus, it is expected that cancer of liver origin would be tightly associated with the changes in metabolic enzymes. Nucleic acid metabolism essentially orchestrates the synthesis and degradation of DNA and RNA. Nucleotides, including purines and pyrimidines, are primarily produced in the liver.^6^ To sustain the proliferation of malignant cells, purine‐ and pyrimidine‐synthesis pathways are often upregulated as a result of increased demand of nucleotides in cancer cells. Thus, the key enzymes involved in these pathways may represent promising anti‐cancer targets.^7^ In this respect, inosine‐5’‐monophosphate dehydrogenase (IMPDH) attracts research interest. This enzyme catalyzes the nicotinamide adenine dinucleotide (NAD^+^)‐dependent oxidation of inosine monophosphate (IMP) to xanthosine monophosphate (XMP),^8^ which is the key step in *de novo* biosynthesis of purines. IMPDH thus controls the size of the guanosine nucleotide pool, which in turn controls many physiological processes, including replication, transcription, and signaling.^9^

In human, *IMPDH* has two isoforms, including *IMPDH1* and *IMPDH2*. They lie on two different chromosomes (chromosomes 7 and 3, respectively), but share 84% amino acid similarity and virtually indistinguishable catalytic activity.^10^ In general, IMPDH1 is thought to be constitutively expressed in most cells,^11^, ^12^ whereas increased IMPDH2 expression has been shown in proliferating and malignant cells.^13^ Especially, the expression and activity of *IMPDH2* are upregulated in various tumors, which are associated with aggressiveness in several experimental cancer models, and poor clinical outcome.^14^, ^15^, ^16^, ^17^ However, the role of IMPDH1 in cancer has not been well studied. In this study, we aim to investigate the expression and function of IMPDH1 in HCC patients and in experimental models.

## 2 MATERIALS AND METHODS

### 2.1 Immunohistochemistry

The tissue microarray (TMA) slides (*n* = 154) of liver tumor tissue were deparaffinized with xylene and rehydrated in graded alcohols for immunohistochemistry (IHC) staining. For antigen retrieval, slides were boiled in citric acid buffer (pH = 6.0) for 14 min. Peroxidase was blocked by using 3% hydrogen peroxide (H_2_O_2_) for 10 min at room temperature. The slides were incubated overnight with the primary antibody against IMPDH1 (1:150, polyclonal antibody from rabbit, Abcam) at 4℃. After being rinsed in PBST, slides were incubated with second antibody (HRP‐conjugated anti‐rabbit, Sigma‐Aldrich) for 1 h at room temperature. Subsequently, 0.05% DAB solution was employed to visualize the staining, and hematoxylin was used for tissue counterstaining. Negative control was carried out by omitting the primary antibody.

For evaluation of the cytoplasmic expression of the enzyme, the percentage of positive cells was scored as: grade 0 for 0%–5%; grade 1 for 5%–30%; grade 2 for 30%–70%; grade 3 for >70%. The staining intensity was graded as: grade 1 for weak; grade 2 for moderate; grade 3 for strong. A final immune‐reactivity score (IRS) was obtained for each case by multiplying the percentage and the intensity values. For example, final score =grade 2 (at the percentage of 30%–70%) × grade 2 (at the intensity of moderate) =4. The scoring was done by three investigators and the differences in scoring were evaluated by Kappa test.

### 2.2 Cell culture and colony‐forming assay

HCC cell line HuH7 and human embryonic kidney epithelial cell line 293T (HEK293T) were cultured in Dulbecco's Modified Eagle's Medium (DMEM) (Invitrogen) supplemented with 10% fetal bovine serum (FBS) (Sigma‐Aldrich) and 100 IU/ml penicillin/streptomycin (Gibco, Bleiswijk). IMPDH1 knockdown (IMPDH1^KD^)cells and control cells were generated by inoculation of lentiviral vectors and subsequently selected and maintained in DMEM medium with 10% FBS, 100 IU/ml penicillin/streptomycin and 2.5 μg/ml puromycin.

Colony‐forming unit (CFU) assay was performed to detect single cell proliferation. Cells were harvested and suspended in medium, 1000 cells were planted into each well of 6‐well plates. Two weeks later, formed colonies were fixed by 70% ethanol and counterstained with hematoxylin and eosin. Colony numbers were counted and the sizes were measured microscopically using Scan Scope software.

### 2.3 Gene knockdown by lentiviral vector delivered short‐hairpin RNA

To generate stable gene knockdown, cells were transduced with lentiviral shRNA vectors. These vectors were obtained from the Erasmus Medical Center for Biomics (the Sigma‐Aldrich TRC library). A vector expressing a mock shRNA that does not target any gene in HCC cells served as control (CTR). The backbone vectors pVSVG, pPMD, and pREV were used to produce lentiviral particles in HEK 293T cells. Because the vectors also express a puromycin resistance gene, transduced cells were subsequently selected by puromycin for three days. After pilot study, the shRNA vectors exerting optimal gene knockdown were selected by RT‐qPCR and Western blot assays. The selected shRNA target sequences were as follows: IMPDH1^KD1^, 5′‐GTGACGTTGAAAGAGGCAAAT‐3′; IMPDH1^KD2^, 5′‐CCAGGATTCATAGAC TTCATA‐3′.

### 2.4 RNA isolation and RT‐qPCR analysis

Total RNA was extracted from cells by using RNA isolation kit (Sigma‐Aldrich). First strand cDNA was synthesized from 1 µg of total RNA using ready‐to‐go first strand beads (GE Healthcare); RT‐qPCR was performed by using Go Taq Real‐Time qPCR mix (Promega); GAPDH was considered as reference gene to normalize target gene expression. Fold changes were determined by using $2^{‐\Delta \Delta C_{T}}$ and normalized to GAPDH. Finally, the fold changes were obtained by converting the logarithmic scale to an exponential scale ($2^{‐\Delta \Delta C_{T}}$). The primers were used as follows: IMPDH1_F, GCACACTGTGGGCGAT; IMPDH1_R, GAGCCACCA CCAGTTCA; IMPDH2_F, TCTTCAACTGCGGAGAC; IMPDH2_R, CTGTAAGCGCCATT GCT; GAPDH_F, GTCTCCTCTGACTTCAACAGCG; GAPDH_R, ACCACCCTGTTGCTGTAGCC AA.

### 2.5 Western blot analysis

Cells were washed with cold PBS gently. A volume of 250 µL of cell lysis buffer containing 0.1 M dithiothreitol was added to each well and incubated for 5 min at 95℃. Proteins (40 μg) were separated by 10% SDS‐PAGE and then transferred to a PVDF membrane (Millipore, Bedford, MA). Membranes were put into blocking buffer at room temperature for 1 h, and then incubated with primary antibodies against IMPDH1 (1:500), IMPDH2 (1:1000), or β‐actin (1:1000, monoclonal antibody from mouse served as the loading standard, Sigma‐Aldrich) overnight at 4℃. After washing, the membranes were incubated with anti‐rabbit or anti‐mouse IRDye‐conjugated secondary antibodies (1:5000, Li‐COR, Lincoln, USA) for 1 hour at room temperature. The membranes were incubated for 1 h at room temperature with anti‐rabbit or anti‐mouse HRP‐conjugated secondary antibodies (1:5000, Bio‐Rad Laboratories). Results were visualized by using the ELC detecting kit (PerkinElmer Inc. MA, USA) and the Tanon 5500 gel imaging system (Tanon Science & Technology Co. Ltd).

### 2.6 HCC xenograft tumor model in immunodeficient mice

The xenograft tumor model in immunodeficient mice was performed in accordance with current prescribed guidelines and under a protocol approved by the Institutional Animal Care and Use Committee of Zhejiang Sci‐Tech University. Mice were bred in SPF environment during the whole experimental period. Balb/c male nude mice were 6–8 weeks (weight: 14–16 g) of age at the time of inoculation. Mice were subcutaneously inoculated with 7.5 million of CTR or IMPDH1^KD^ HuH7 cells into the left and right back, respectively. Four weeks later, mice were sacrificed and tumors were harvested and weighed.

### 2.7 Genome‐wide transcriptomic profiling and data analysis

A total amount of 2 μg RNA per sample was used as input material for the RNA sample preparations. Sequencing libraries were generated using VAHTSTM mRNA‐seq V2 Library Prep Kit for Illumina^®^ following the manufacturer's recommendations. Gene expression values of the transcripts were computed by StringTie (version 1.3.3b). DESeq2 (version 1.12.4) was used to determine differentially expressed genes (DEGs) between two samples. Genes were considered as significant differentially expressed if q‐value <0.001 and fold change >2. Gene Ontology (GO) and Kyoto Encyclopedia of Genes and Genomes (KEGG) database were performed to identify which DEGs were significantly enriched. GO terms and KEGG pathway with false discovery rate (q‐value) <0.05 were considered significantly altered.

### 2.8 Statistical analysis

Statistical analysis was performed by using Kappa test and Kaplan‐Meier survival analysis in IBM SPSS Statistical. Mann‐Whitney test and Wilcoxon matched pairs test were adopted by using GraphPad software. Differences were significant at *p* < 0.05.

## 3 RESULTS

### 3.1 IMPDH1 is upregulated in patient HCC tumors and higher expression in tumor is associated with better clinical outcome

**FIGURE 1:**
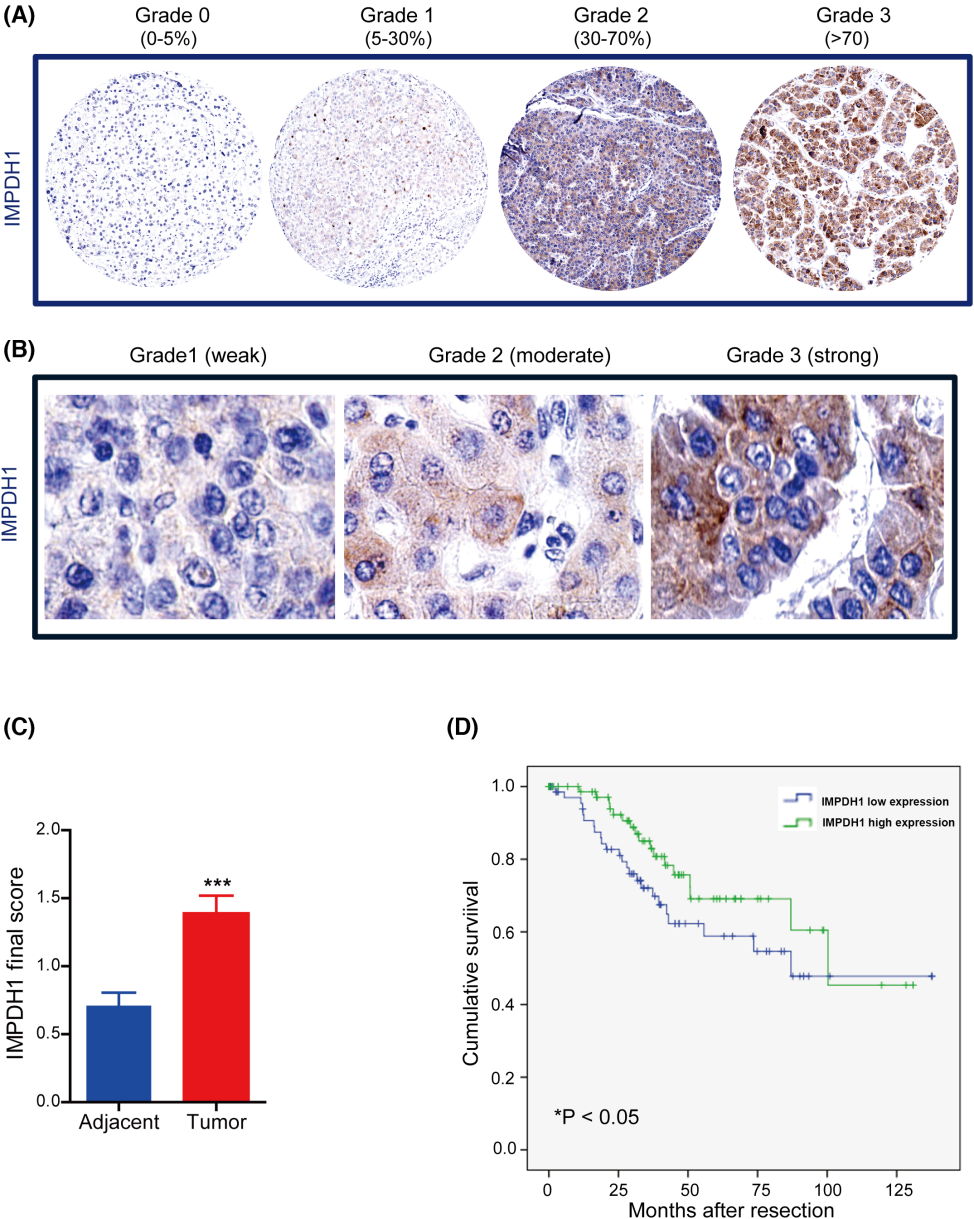
Expression of IMPDH1 is upregulated in HCC tumors, and high expression is significantly associated with better clinical outcome in HCC patients. (A) The levels of IMPDH1 based on the percentages of positivity range from grade 0 (<5%), grade 1 (5%–30%), grade 2 (30%–70%) to grade 3 (>70%) both in the HCC tumors and in their adjacent sites (200 x magnification); (B) The levels of IMPDH1 based on intensity range from grade 1 (weak) to grade 2 (moderate) to grade 3 (strong) (400 x magnification); (C) The expression of IMPDH1 in tumor is significantly higher than in the adjacent liver tissue (Wilcoxon matched pairs test, *n* = 154, ***p < 0.001); (D) Patients with higher expression of IMPDH1 in tumors have longer survival (Kaplan‐Meier, *n* = 151, *p < 0.05) (Green line, final IRS scores 1–9; Blue line, final IRS scores 0–0.99, cut‐off = 0.99)

To investigate the expression of IMPDH1 in HCC, immunohistochemistry staining of TMA slides from HCC patients was performed (Figure 1A and B). We found that the cytoplasmic expression of IMPDH1 protein in tumors was significantly higher than in adjacent tumor‐free liver tissues (*n* = 154, *p* < 0.001, Figure 1C). Surprisingly, higher IMPDH1 protein level in tumors was associated with better clinical outcome in HCC patients (*n* = 151, *p* < 0.05, Figure 1D). Given the significance of IMPDH1 in HCC patients, we next investigated the function of this protein in experimental HCC models.

### 3.2 Loss of IMPDH1 inhibits colony formation, tumor initiation, and growth

**FIGURE 2:**
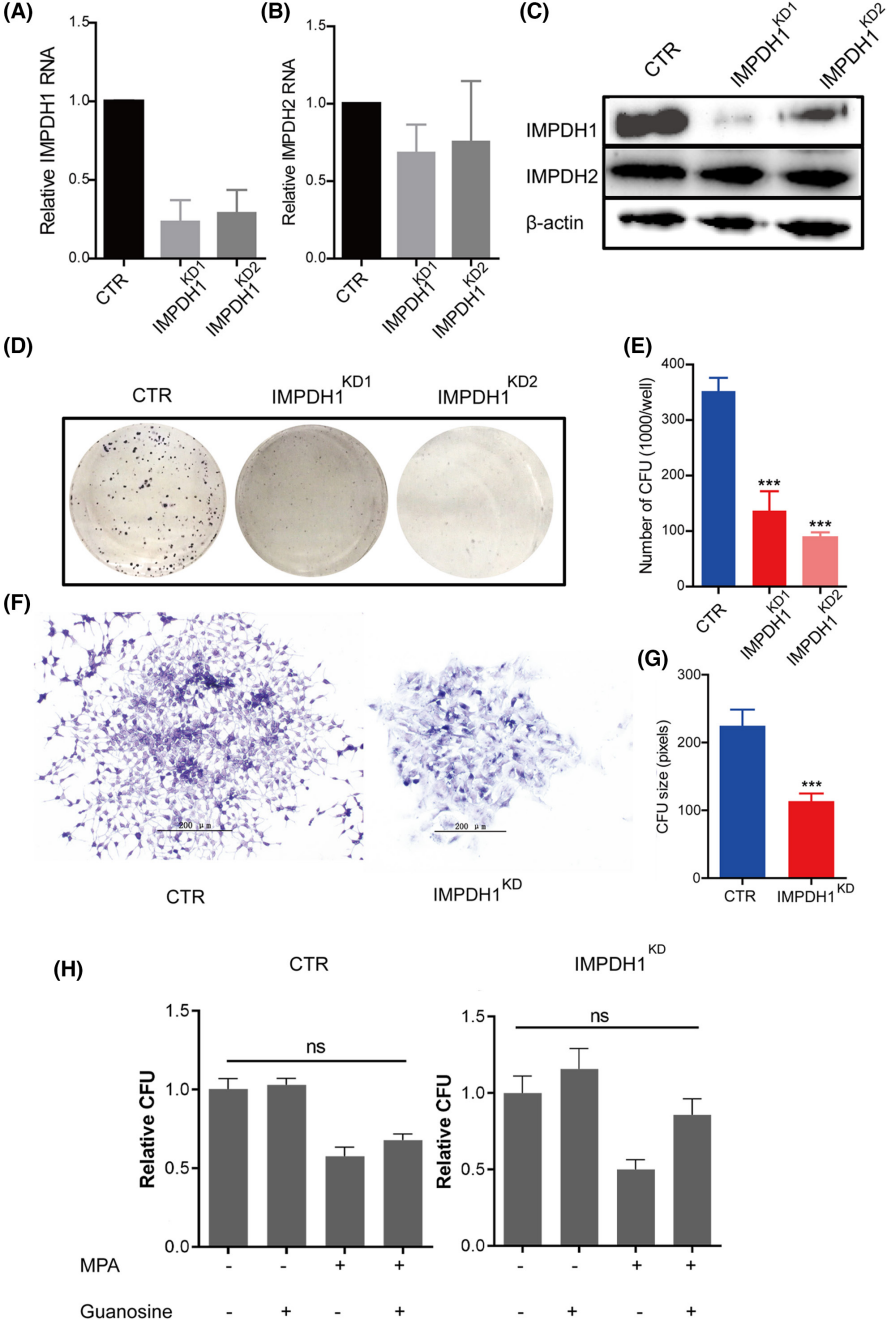
Loss of IMPDH1 decreases colony formation in vitro. (A) The mRNA level of IMPDH1 is decreased in IMPDH1^KD^ HuH7 cells (clone1 and clone2 of IMPDH1 knockdown cells are marked as IMPDH1^KD1^ and IMPDH1^KD2^); (B) The mRNA level of IMPDH2 is not affected in IMPDH^KD^cells; (C) In IMPDH1^KD^ cells, the protein level of IMPDH1 is dramatically decreased; while the level of IMPDH2 is not affected. (D) Colony appearance of CTR and IMPDH1^KD^ cells; (E) IMPDH1^KD^ cells are inferior in CFU compared with CTR cells (Mann‐Whitney test, *n* = 7, ***p < 0.001); (F) Colony appearance of CTR and IMPDH1^KD^ cells under the microscope (200 x magnification); (G) Loss of IMPDH1 impairs the size of colony growth (Mann‐Whitney test, *n* = 10, ***p < 0.001); (H) CFU assay showed the effects of MPA or/and guanosine on IMPDH1^KD^ cells. The concentrations of MPA and guanosine are 3 μM and 25 μM, respectively. (Mann‐Whitney test, *n* ≥ 6)

For functional characterization, we established stable IMPDH1 knockdown HuH7 cells by lentiviral RNAi approach. Successful knockdown was confirmed at both mRNA and protein levels without affecting the expression of IMPDH2 (Figure 2A, B and C).

Next, CFU assay was performed to evaluate the effects on proliferation at single cell level. We observed that downregulation of IMPDH1 significantly inhibited the CFU capability of HuH7 cell (CTR vs IMPDH1^KD1^ and IMPDH1^KD2^, 352.00 ± 24.26 vs 136.78 ± 34.97 and 88.86 ± 9.13 colonies per 1000 cells, mean ±SEM, *n* = 7, *p* < 0.001, Figure 2D and E). Accordingly, the size of CFU was significantly smaller in IMPDH1^KD^ cells when compared with CTR cells (CTR vs IMPDH1^KD^, 224.90 ± 23.77 vs 113.50 ± 26.32 pixels, mean ±SEM, *n* = 10, *p* < 0.001, Figure 2F and G).

Mycophenolic acid (MPA) acts as a nonnucleoside, noncompetitive, reversible inhibitor of IMPDH, and is currently widely used for prevention of allograft rejection.^10^ It plays a role through depletion of guanine nucleotide pools by inhibition of IMPDH.^18^ Herein, we observed the response of control cell and IMPDH1^KD^ HuH7 cell to MPA and/or exogenous guanosine treatment. The results showed that both of the cells displayed impaired CFU activity when treated with MPA, while guanosine supplementation reversed this phenomenon (Figure 2H). However, this effect was not distinct.

**FIGURE 3:**
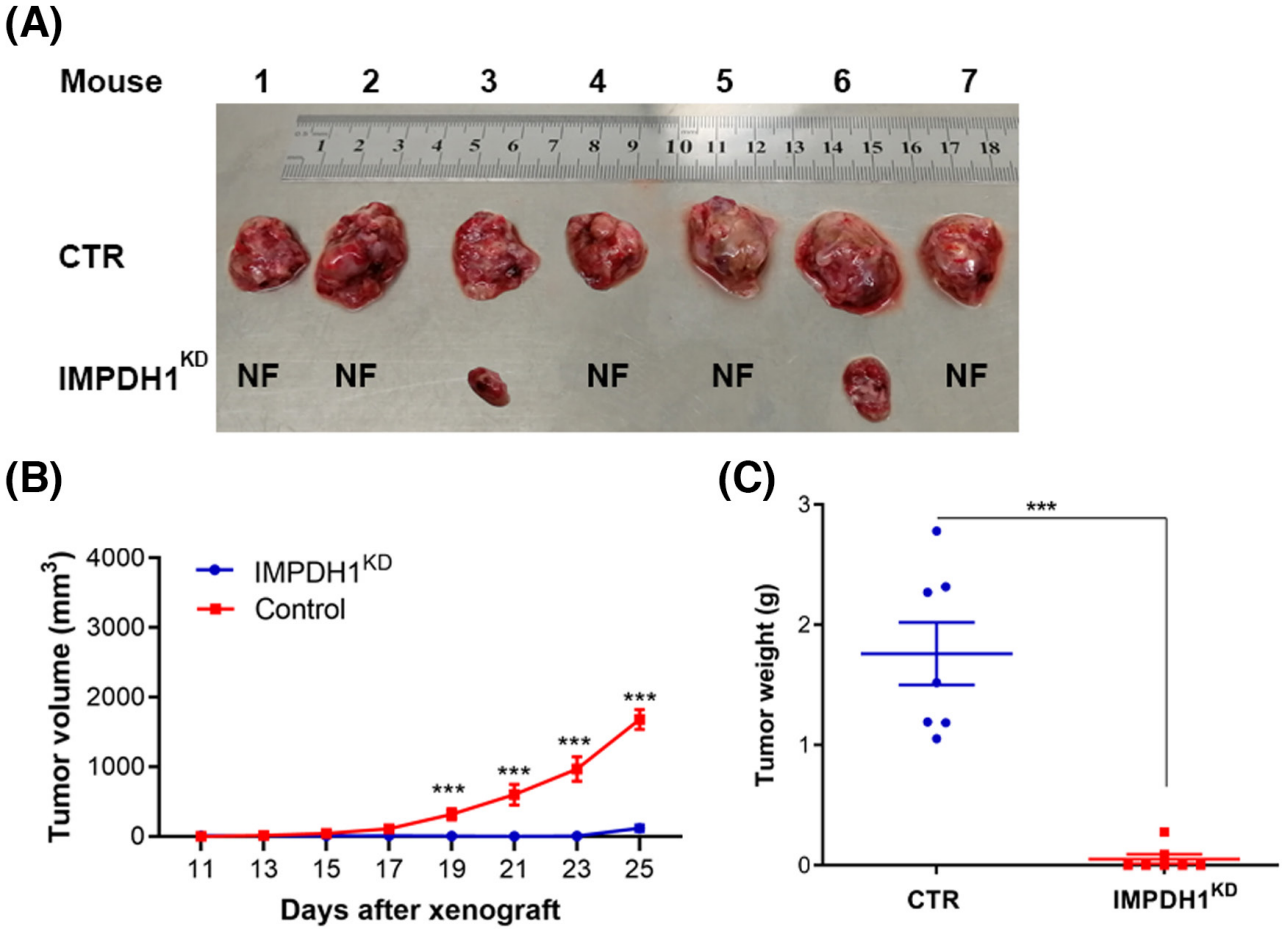
Loss of IMPDH1 inhibits tumor initiation and growth in mice. (A) Appearance of tumors from the xenograft models; (B) The growth speed of tumors originating from IMPDH1^KD^ cells is slower than that of the tumors originating from CTR cells; (C) Tumors originating from IMPDH1^KD^ cells are significantly lighter than the tumors originating from CTR cells (Wilcoxon matched pairs test, *n* = 9, ***p < 0.001)

To assess this observation in vivo, we performed xenografting of HCC cells in immunodeficient mice. We compared the capacity of tumor initiation between CTR and IMPDH1^KD^ cells in vivo. To this end, 7.5 million CTR or IMPDH1^KD^ HuH7 cells were injected subcutaneously into immunodeficient mice on the left or right side of the back, respectively. As shown in Figure 3A, IMPDH1^KD^ cells were inferior in tumor initiation (CTR vs IMPDH1^KD^, 9/9 vs 2/9) and tumor growth (tumor weight: CTR vs IMPDH1^KD^, 2.12 ± 0.32 vs 0.04 ± 0.03 g, mean ±SEM, *n* = 9, *p* < 0.001, Figure 3B and C). Thus, loss of IMPDH1 leads to suppress tumor growth and progression, and these results demonstrate that IMPDH1 molecules appear to sustain tumor growth and aggressiveness.

### 3.3 Silencing of IMPDH1 regulates multiple molecular pathways

**FIGURE 4:**
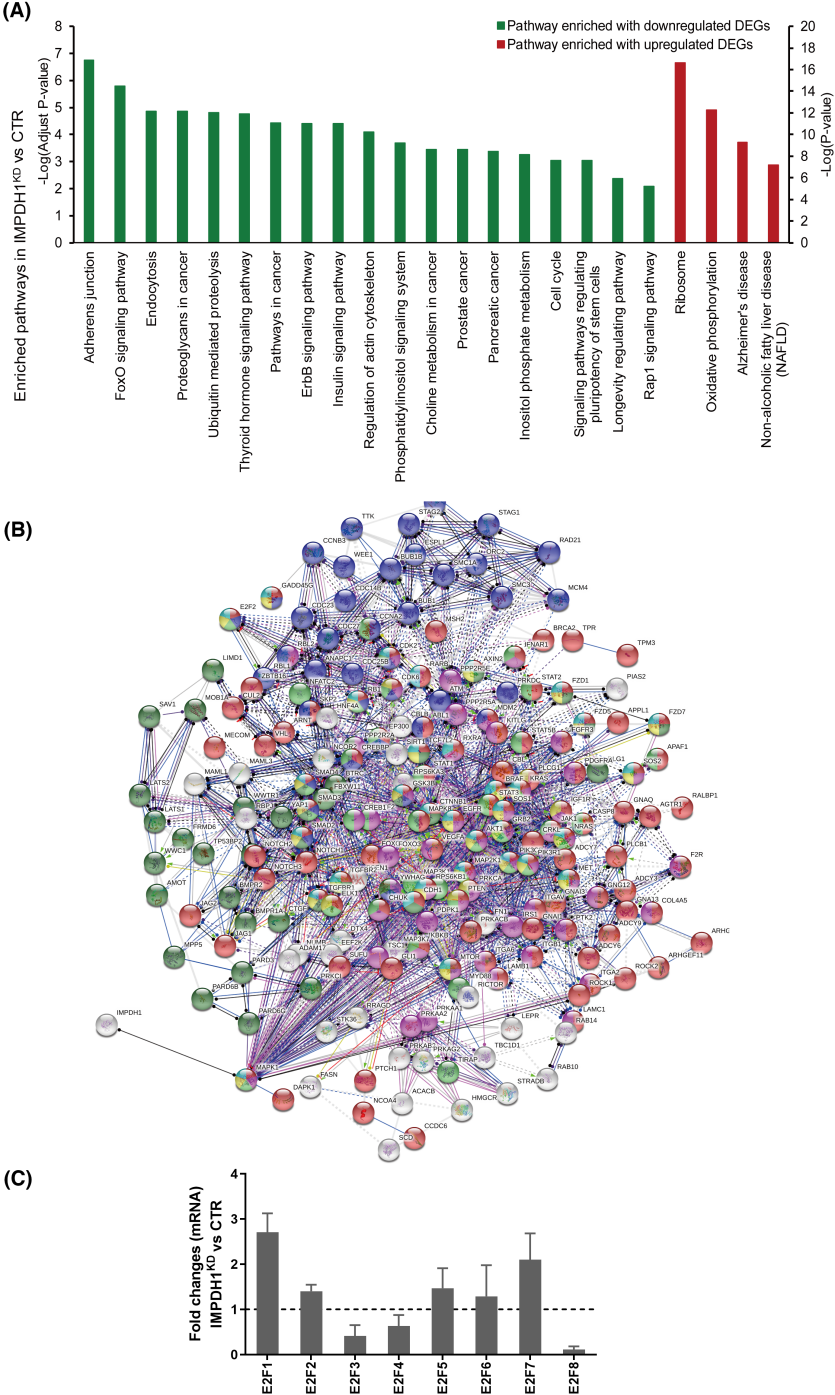
Loss of IMPDH1 regulates molecular pathways. (A) Significantly altered KEGG pathways in IMPDH1^KD^ cells; (B) A subnetwork composing of 205 nodes and 1568 edges was generated using differently expressed genes in IMPDH1^KD^ cells. Pathway enrichment analysis shows that the subnetwork is mainly enriched in cancer‐related pathways (Red ball represents pathways in cancer, yellow ball represents hepatocellular carcinoma, pink ball represents PI3K/Akt signaling pathway, purple ball represents cell cycle, dark green ball represents hippo signaling pathway, green ball represents hepatitis B, blue ball represents gastric cancer, and brown ball represents p53 signaling pathways). (C) q‐PCR shows that the expression levels of *E2F* family genes were changed in IMPDH1^KD^ cells

To map the effects of IMPDH1 on cellular signaling pathways, a genome‐wide transcriptomic analysis was performed in IMPDH1^KD^ HuH7 cell models. A total of 9250 genes were altered by >2 folds upon silencing of IMPDH1 compared to control cells. The significantly altered genes were functionally annotated to identify the molecular pathways. Among the prebuilt KEGG pathways, at least 46 pathways were significantly altered after loss of IMPDH1 (Table S1, downregulated DEGs enriched pathways with adjust *p* value <0.05, upregulated DEGs enriched pathways with *p* value <0.05), and the most significantly altered processes are shown in Figure 4A (adjust *p* value < 0.01 or *p* < 0.01). Protein‐protein interaction analysis further revealed that IMPDH1 (Figure 4B and Table S2) was involved in many cancer‐related pathways.

Although IMPDH is largely cytoplasmic, a previous study has shown that IMPDH could be transferred to the nucleus to bind with RNA and / or DNA.^19^ It has been reported that drosophila IMPDH can act as a DNA‐binding transcriptional repressor. Triggered by cell cycling or oxidative stress, IMPDH accumulates to suppress the expression of E2F genes, the key drivers of cell proliferation, and thus inhibits the growth of drosophila cells.^20^ Thus, we detected the mRNA level of E2F family members in IMPDH1^KD^ HuH7 cells. The results identified that the expression of E2F1 and E2F7 was significantly enhanced in IMPDH1 silenced cells (Figure 4C).

## 4 DISCUSSION

In general, IMPDH1 is constitutively expressed in most cells/tissues.^21^ It has been identified as a target gene of an important oncogenic miRNA (miRNA‐19a), and exogenous expression of this gene has no effect on cancer cell growth.^22^ We found that the expression of IMPDH1 is significantly higher in tumor of HCC patients when compared to adjacent liver tissue. Functionally, loss of IMPDH1 in HCC cells impaired colony formation in vitro and tumor initiation/growth in mice,suggesting that higher expression of IMPDH1 sustained tumor growth and aggressiveness. The online database (http://gepia.cancer‐pku.cn/index.html) reported that the expression of IMPDH1 is higher in tumors (*n* = 369) than in normal tissues (*n* = 160, Figure S1A) and the expression of IMPDH1 is associated with HCC aggressiveness (Figure S1B), which are consistent with our results. However, it is discrepant that high IMPDH1 expression is correlated with poor clinical outcome (Figure S1C). One possible reason may be the data source, in view of the complexity of etiology and pathogenesis of HCC patients involved, and our TMA slides are from less European patients who received surgical resection and treatment.

Chong et al. reported that PI3k‐E2F axis plays a critical role in the proliferation of HCC by regulating purine biosynthetic enzyme, and *IMPDH* acts as an E2F1‐dependent target genes.^23^ However, they did not detect the response of E2F family members to IMPDH silencing. Our result identified that high expression of E2F1 and E2F7 were closely related to IMPDH1 silence, implying a feedback loop between E2F family and IMPDH. This may provide insights for IMPDH1 research.

In the setting of liver transplantation, a substantial proportion of patients are transplanted for curing HCC. However, prevention of tumor recurrence is the major challenge for achieving this goal. Thus, an ideal immunosuppressant regimen would possess immunosuppressive and anti‐tumor functions simultaneously. As non‐nucleoside, non‐competitive, and reversible inhibitor of IMPDH, MPA has been widely used in the clinic for decades. Chong et al. reported that both of the growth of xenograft tumors with high IMPDHs expression and the HuH7 cells with IMPDHs silencing could be inhibited by MPA, while the effect of MPA on the frequency of tumor formation in DEN‐induced mouse model was not observed.^23^ Our single cell colony‐forming unit assay further demonstrated that IMPDH1 silencing HuH7 cell exhibited obscure distinction to MPA treatment when compared with control cell. These implying multiple anti‐cancer mechanisms of MPA may be involved. A recent retrospective analysis has indicated that use of MPA is associated with lower risk of tumor recurrence and better survival in HCC patients after liver transplantation,^24^ thus warranting future prospective clinical trials to confirm these findings.

In conclusion, the expression of IMPDH1 is upregulated in the tumor tissues. In experimental models, we have demonstrated that IMPDH1 sustains HCC. These findings offer insights into the distinct roles of IMPDH1 in HCC and bear important implications for future IMPDH‐targeted anti‐cancer drug development.

## CONFLICT OF INTEREST

The authors declare no conflict of interest.

## AUTHOR CONTRIBUTIONS

X.J and Y.W performed experiments and data analysis; Y.C and H.K were responsible for acquisition of data and interpretation of data; X.J was responsible for and drafting of the manuscript; Y.L and Z.M were involved in project discussion and data analysis/interpretation; K.C was responsible for study design, study supervision, and critical revision of the manuscript.

## Supporting information

Supplementary Material

## Data Availability

The data that support the findings of this study are available from the corresponding author upon reasonable request.

## References

[1] Hanahan D , Weinberg RA . Hallmarks of cancer: the next generation. Cell. 2011;144(5):646‐674. 21376230 10.1016/j.cell.2011.02.013

[2] Pavlova NN , Thompson CB . The emerging hallmarks of cancer metabolism. Cell Metab. 2016;23(1):27‐47. 26771115 10.1016/j.cmet.2015.12.006 PMC4715268

[3] Cairns RA , Harris IS , Mak TW . Regulation of cancer cell metabolism. Nat Rev Cancer. 2011;11(2):85‐95. 21258394 10.1038/nrc2981

[4] Ward PS , Thompson CB . Metabolic reprogramming: a cancer hallmark even warburg did not anticipate. Cancer Cell. 2012;21(3):297‐308. 22439925 10.1016/j.ccr.2012.02.014 PMC3311998

[5] Cheong H , Lu C , Lindsten T , Thompson CB . Therapeutic targets in cancer cell metabolism and autophagy. Nat Biotechnol. 2012;30(7):671‐678. 22781696 10.1038/nbt.2285 PMC3876738

[6] Hsu PP , Sabatini DM . Cancer cell metabolism: Warburg and beyond. Cell. 2008;134(5):703‐707. 18775299 10.1016/j.cell.2008.08.021

[7] Warburg O . On the origin of cancer cells. Science. 1956;123(3191):309‐314. 13298683 10.1126/science.123.3191.309

[8] Shu Q , Nair V . Inosine monophosphate dehydrogenase (IMPDH) as a target in drug discovery. Med Res Rev. 2008;28(2):219‐232. 17480004 10.1002/med.20104

[9] Weber G , Nakamura H , Natsumeda Y , Szekeres T , Nagai M . Regulation of GTP biosynthesis. Adv Enzyme Regul. 1992;32:57‐69. 1353938 10.1016/0065-2571(92)90008-n

[10] Sintchak MD , Fleming MA , Futer O , et al. Structure and mechanism of inosine monophosphate dehydrogenase in complex with the immunosuppressant mycophenolic acid. Cell. 1996;85(6):921‐930. 8681386 10.1016/s0092-8674(00)81275-1

[11] Kennan A , Aherne A , Palfi A , et al. Identification of an IMPDH1 mutation in autosomal dominant retinitis pigmentosa (RP10) revealed following comparative microarray analysis of transcripts derived from retinas of wild‐type and Rho(‐/‐) mice. Hum Mol Genet. 2002;11(5):547‐557. 11875049 10.1093/hmg/11.5.547

[12] Bowne SJ , Sullivan LS , Blanton SH , et al. Mutations in the inosine monophosphate dehydrogenase 1 gene (IMPDH1) cause the RP10 form of autosomal dominant retinitis pigmentosa. Hum Mol Genet. 2002;11(5):559‐568. 11875050 10.1093/hmg/11.5.559 PMC2585828

[13] Collart FR , Chubb CB , Mirkin BL , Huberman E . Increased inosine‐5'‐phosphate dehydrogenase gene expression in solid tumor tissues and tumor cell lines. Cancer Res. 1992;52(20):5826‐5828. 1356621 10.2172/10148922

[14] Floryk D , Tollaksen SL , Giometti CS , Huberman E . Differentiation of human prostate cancer PC‐3 cells induced by inhibitors of inosine 5'‐monophosphate dehydrogenase. Cancer Res. 2004;64(24):9049‐9056. 15604271 10.1158/0008-5472.CAN-04-1553

[15] Moosavi MA , Yazdanparast R , Sanati MH , Nejad AS . 3‐Hydrogenkwadaphnin targets inosine 5'‐monophosphate dehydrogenase and triggers post‐G1 arrest apoptosis in human leukemia cell lines. Int J Biochem Cell Biol. 2005;37(11):2366‐2379. 16084123 10.1016/j.biocel.2005.04.020

[16] Fox CB , Wayment JR , Myers GA , Endicott SK , Harris JM . Single‐molecule fluorescence imaging of peptide binding to supported lipid bilayers. Anal Chem. 2009;81(13):5130‐5138. 19480398 10.1021/ac9007682

[17] He Y , Mou Z , Li W , et al. Identification of IMPDH2 as a tumor‐associated antigen in colorectal cancer using immunoproteomics analysis. Int J Colorectal Dis. 2009;24(11):1271‐1279. 19597826 10.1007/s00384-009-0759-2

[18] Carr SF , Papp E , Wu JC , Natsumeda Y . Characterization of human type I and type II IMP dehydrogenases. J Biol Chem. 1993;268(36):27286‐27290. 7903306

[19] McLean JE , Hamaguchi N , Belenky P , Mortimer SE , Stanton M , Hedstrom L . Inosine 5'‐monophosphate dehydrogenase binds nucleic acids in vitro and in vivo. Biochem J. 2004;379(Pt 2):243‐251. 14766016 10.1042/BJ20031585 PMC1224093

[20] Kozhevnikova EN , Knaap JA , Pindyurin AV , et al. Metabolic enzyme IMPDH is also a transcription factor regulated by cellular state. Mol Cell. 2012;47(1):133‐139. 22658723 10.1016/j.molcel.2012.04.030

[21] Hedstrom L . IMP dehydrogenase: structure, mechanism, and inhibition. Chem Rev. 2009;109(7):2903‐2928. 19480389 10.1021/cr900021w PMC2737513

[22] Ouchida M , Kanzaki H , Ito S , et al. Novel direct targets of miR‐19a identified in breast cancer cells by a quantitative proteomic approach. PLoS One. 2012;7(8):e44095. 22952885 10.1371/journal.pone.0044095 PMC3431339

[23] Chong YC , Toh TB , Chan Z , et al. Targeted Inhibition of purine metabolism is effective in suppressing hepatocellular carcinoma progression. Hepatol Commun. 2020;4(9):1362‐1381. 32923839 10.1002/hep4.1559 PMC7471427

[24] Chen K , Sheng J , Ma B , et al. Suppression of hepatocellular carcinoma by mycophenolic acid in experimental models and in patients. Transplantation. 2019;103(5):929‐937. 30747839 10.1097/TP.0000000000002647

