# Supplementary material for: Inosine monophosphate dehydrogenase type1 sustains tumor growth in hepatocellular carcinoma
Source: J Clin Lab Anal. 2022 Apr 11;36(5):e24416. doi: 10.1002/jcla.24416 (PMC9102537; doi:10.1002/jcla.24416)
Supplement: Supplementary file 1 — Supplementary Material [file JCLA-36-e24416-s001.docx]

**Supplementary information for**

**Inosine monophosphate dehydrogenase type1 sustains tumor growth**

**in hepatocellular carcinoma**

**Supplementary figure 1**

**
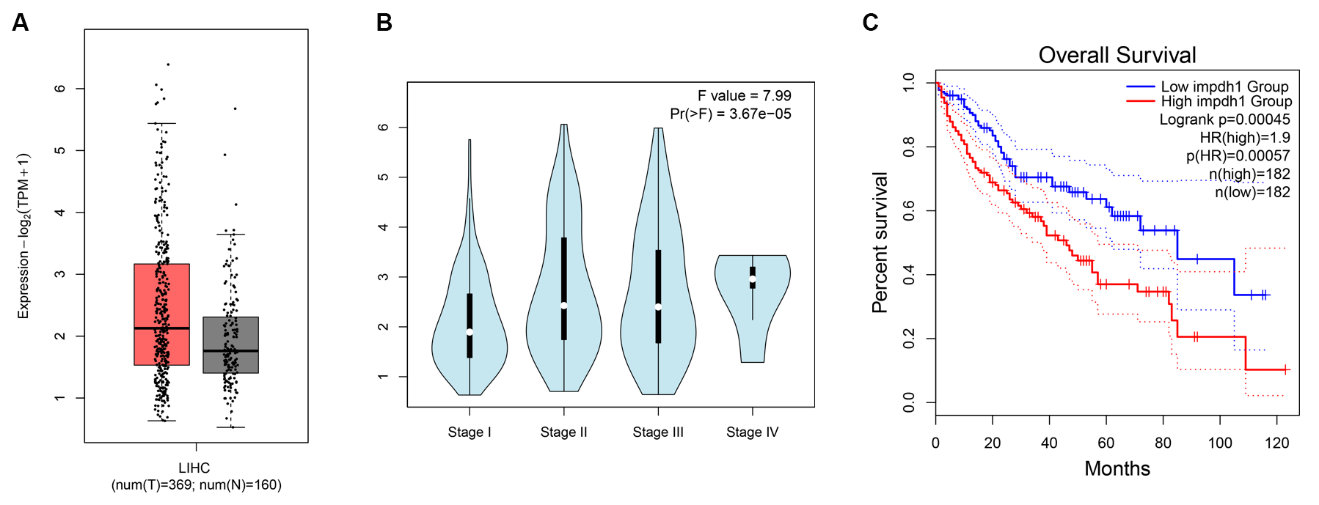
**

Fig S1. The mRNA expression of IMPDHs positively associated with the progression of HCC patient (http://gepia.cancer-pku.cn/index.html). The levels of IMPDH1 (A) are higher in tumor tissue than in normal tissue; Higher expressions of IMPDH1 (B) are detected in stage IV HCC; (C) HCC patient with high IMPDH1 expression shows a poor clinical outcome.

**Supplementary Table I**

**Pathway enrichment analysis of up/down regulated DEGs in**

**IMPDH1^KD^ vs CTR cells**

| **Regulated DEGs** | **KEGG ID** | **KEGG term** | **Overlap Genes** | **P-value** | **Adjusted P-value** |
| --- | --- | --- | --- | --- | --- |
| Down | hsa04520 | Adherens junction | 35/74 | 6.55E-10 | 1.82E-07* |
| Down | hsa04068 | FoxO signaling pathway | 49/133 | 1.15E-08 | 1.60E-06* |
| Down | hsa04144 | Endocytosis | 76/259 | 1.54E-07 | 1.37E-05* |
| Down | hsa05205 | Proteoglycans in cancer | 63/203 | 1.98E-07 | 1.37E-05* |
| Down | hsa04120 | Ubiquitin mediated proteolysis | 47/137 | 2.75E-07 | 1.53E-05* |
| Down | hsa04919 | Thyroid hormone signaling pathway | 42/118 | 3.74E-07 | 1.73E-05* |
| Down | hsa05200 | Pathways in cancer | 103/397 | 9.38E-07 | 3.73E-05* |
| Down | hsa04012 | ErbB signaling pathway | 33/87 | 1.27E-06 | 3.91E-05* |
| Down | hsa04910 | Insulin signaling pathway | 46/139 | 1.17E-06 | 3.91E-05* |
| Down | hsa04810 | Regulation of actin cytoskeleton | 62/214 | 3.28E-06 | 8.29E-05* |
| Down | hsa04070 | Phosphatidylinositol signaling system | 34/98 | 8.87E-06 | 2.05E-04* |
| Down | hsa04213 | Longevity regulating pathway - multiple species | 25/64 | 1.31E-05 | 2.81E-04* |
| Down | hsa05231 | Choline metabolism in cancer | 34/101 | 1.85E-05 | 3.67E-04* |
| Down | hsa05215 | Prostate cancer | 31/89 | 1.98E-05 | 3.68E-04* |
| Down | hsa05212 | Pancreatic cancer | 25/66 | 2.45E-05 | 4.26E-04* |
| Down | hsa00562 | Inositol phosphate metabolism | 26/71 | 3.38E-05 | 5.46E-04* |
| Down | hsa04110 | Cell cycle | 38/124 | 6.61E-05 | 9.19E-04* |
| Down | hsa04550 | Signaling pathways regulating pluripotency of stem cells | 42/142 | 7.03E-05 | 9.31E-04* |
| Down | hsa04211 | Longevity regulating pathway - mammal | 29/94 | 4.06E-04 | 4.18E-03* |
| Down | hsa04015 | Rap1 signaling pathway | 53/211 | 8.60E-04 | 8.25E-03* |
| Down | hsa05161 | Hepatitis B | 39/146 | 1.17E-03 | 1.05E-02* |
| Down | hsa00310 | Lysine degradation | 18/52 | 1.14E-03 | 1.05E-02* |
| Down | hsa04152 | AMPK signaling pathway | 34/124 | 1.45E-03 | 1.22E-02* |
| Down | hsa04540 | Gap junction | 26/88 | 1.60E-03 | 1.31E-02* |
| Down | hsa04931 | Insulin resistance | 30/109 | 2.50E-03 | 1.88E-02* |
| Down | hsa04912 | GnRH signaling pathway | 26/91 | 2.69E-03 | 1.96E-02* |
| Down | hsa04150 | mTOR signaling pathway | 19/60 | 2.75E-03 | 1.96E-02* |
| Down | hsa05131 | Shigellosis | 20/65 | 3.15E-03 | 2.19E-02* |
| Down | hsa05210 | Colorectal cancer | 19/62 | 4.14E-03 | 2.74E-02* |
| Down | hsa05219 | Bladder cancer | 14/41 | 4.51E-03 | 2.91E-02* |
| Down | hsa04390 | Hippo signaling pathway | 38/153 | 5.25E-03 | 3.28E-02* |
| Down | hsa05222 | Small cell lung cancer | 24/86 | 5.31E-03 | 3.28E-02* |
| Down | hsa04072 | Phospholipase D signaling pathway | 36/144 | 5.78E-03 | 3.42E-02* |
| Down | hsa04530 | Tight junction | 35/139 | 5.71E-03 | 3.42E-02* |
| Down | hsa04933 | AGE-RAGE signaling pathway in diabetic complications | 27/101 | 6.13E-03 | 3.45E-02* |
| Down | hsa04722 | Neurotrophin signaling pathway | 31/120 | 6.03E-03 | 3.45E-02* |
| Down | hsa04666 | Fc gamma R-mediated phagocytosis | 25/93 | 7.56E-03 | 4.04E-02* |
| Down | hsa04330 | Notch signaling pathway | 15/48 | 8.43E-03 | 4.42E-02* |
| Up | hsa03010 | Ribosome | 29/137 | 2.25E-17^#^ | 4.09E-15 |
| Up | hsa00190 | Oxidative phosphorylation | 24/133 | 5.44E-13^#^ | 4.95E-11 |
| Up | hsa05010 | Alzheimer's disease | 23/168 | 5.50E-10^#^ | 2.50E-08 |
| Up | hsa04932 | Non-alcoholic fatty liver disease (NAFLD) | 19/151 | 7.00E-08^#^ | 2.12E-06 |
| Up | hsa03020 | RNA polymerase | 4/32 | 1.28E-02^#^ | 3.32E-01 |
| Up | hsa00480 | Glutathione metabolism | 5/52 | 1.64E-02^#^ | 3.73E-01 |
| Up | hsa01100 | Metabolic pathways | 48/1239 | 2.13E-02^#^ | 4.31E-01 |
| Up | hsa00240 | Pyrimidine metabolism | 7/105 | 3.16E-02^#^ | 5.75E-01 |

DEGs: Differentially expressed genes. * Represents Adjusted P-value<0.05. ^#^ Represents P-value<0.05.

**Supplementary Table II**

**Pathway enrichment analysis of DEGs in IMPDH1^KD^ vs CTR cells included in PPI networks (Adjust P-value<10^-29^)**

| **KEGG ID** | **KEGG term** | **Count in gene set** | **false discovery rate** |
| --- | --- | --- | --- |
| hsa05200 | Pathways in cancer | 110 of 515 | 2.89E-108 |
| hsa05165 | Human papillomavirus infection | 69 of 317 | 1.5E-64 |
| hsa04068 | FoxO signaling pathway | 41 of 130 | 1.1E-42 |
| hsa04151 | PI3K-Akt signaling pathway | 53 of 348 | 1.32E-41 |
| hsa04110 | Cell cycle | 39 of 123 | 8.37E-41 |
| hsa05161 | Hepatitis B | 39 of 142 | 7.93E-39 |
| hsa04390 | Hippo signaling pathway | 38 of 152 | 1.56E-36 |
| hsa04152 | AMPK signaling pathway | 34 of 120 | 3.48E-34 |
| hsa05166 | HTLV-I infection | 41 of 250 | 3.83E-33 |
| hsa05205 | Proteoglycansincancer | 38 of 195 | 3.85E-33 |
| hsa05225 | Hepatocellular carcinoma | 36 of 163 | 4.53E-33 |
| hsa05226 | Gastric cancer | 34 of 147 | 8.38E-32 |
| hsa05206 | MicroRNAs in cancer | 34 of 149 | 1.15E-31 |
| hsa01522 | Endocrine resistance | 30 of 95 | 1.74E-31 |
| hsa05215 | Prostate cancer | 30 of 97 | 2.74E-31 |
| hsa05212 | Pancreatic cancer | 28 of 74 | 3.12E-31 |
| hsa05224 | Breast cancer | 33 of 147 | 1.26E-30 |
| hsa04910 | Insulin signaling pathway | 32 of 134 | 1.93E-30 |
